# Efficacious, safe, and stable inhibition of corneal neovascularization by AAV-vectored anti-VEGF therapeutics

**DOI:** 10.1016/j.omtm.2021.06.007

**Published:** 2021-06-24

**Authors:** Wenqi Su, Shuo Sun, Bo Tian, Phillip W.L. Tai, Yongwen Luo, Jihye Ko, Wei Zhan, Xiao Ke, Qiang Zheng, Xiaorong Li, Hua Yan, Guangping Gao, Haijiang Lin

**Affiliations:** 1 Department of Ophthalmology, Tianjin Medical University General Hospital, Tianjin 300052, China; 2 Department of Ophthalmology and Visual Sciences, University of Massachusetts Medical School, Worcester, MA 01655, USA; 3 Tianjin Key Laboratory of Retinal Functions and Diseases, Tianjin International Joint Research and Development Centre of Ophthalmology and Vision Science, Eye Institute and School of Optometry, Tianjin Medical University Eye Hospital, Tianjin 300384, China; 4 Horae Gene Therapy Center, University of Massachusetts Medical School, Worcester, MA 01605, USA; 5 Department of Microbiology and Physiological Systems, University of Massachusetts Medical School, Worcester, MA 01605, USA; 6 College of Veterinary Medicine, South China Agricultural University, No. 483, Wushan Road, Guangzhou 510642, China; 7 Viral Vector Core, University of Massachusetts Medical School, Worcester, MA 01605, USA; 8 Chengdu Kanghong Pharmaceutical Group, 36 Shuxi Rd., Jinniu District, Chengdu, Sichuan 610036, China; 9 Li Weibo Institute for Rare Diseases Research, University of Massachusetts Medical School, Worcester, MA 01605, USA

**Keywords:** corneal neovascularization, recombinant adeno-associated virus, anti-VEGF, gene therapy

## Abstract

Corneal neovascularization (CoNV) leads to visual impairment, affecting over 1.4 million people in the United States per year. It is caused by a variety of pathologies, such as inflammation, hypoxia, and limbal barrier dysfunction. Injection of the anti-vascular endothelial growth factor (VEGF) drug KH902 (conbercept) can inhibit CoNV but requires repeated dosing that produces associated side effects, such as cornea scar. To explore more efficacious and long-lasting treatment of CoNV, we employed recombinant adeno-associated virus (rAAV)2 and rAAV8 vectors to mediate KH902 expression via a single intrastromal injection and investigated its anti-angiogenic effects and safety in both alkali-burn- and suture-induced CoNV mouse models. Our results showed that rAAV-mediated *KH902* mRNA expression in the cornea was sustained for at least 3 months after a single intrastromal injection. Moreover, the expression level of rAAV8-*KH902* far exceeded that of rAAV2-*KH902*. A single-dose rAAV8-*KH902* treatment at 8 × 10^8^ genome copies (GCs) per cornea dramatically inhibited CoNV for an extended period of time in mouse CoNV models without adverse events, whereas the inhibition of CoNV by a single intrastromal administration of the conbercept drug lasted for only 10−14 days. Overall, our study demonstrated that the treatment of CoNV with a single dose of rAAV8-*KH902* via intrastromal administration was safe, effective, and long lasting, representing a novel therapeutic strategy for CoNV.

## Graphical abstract

**Figure:**
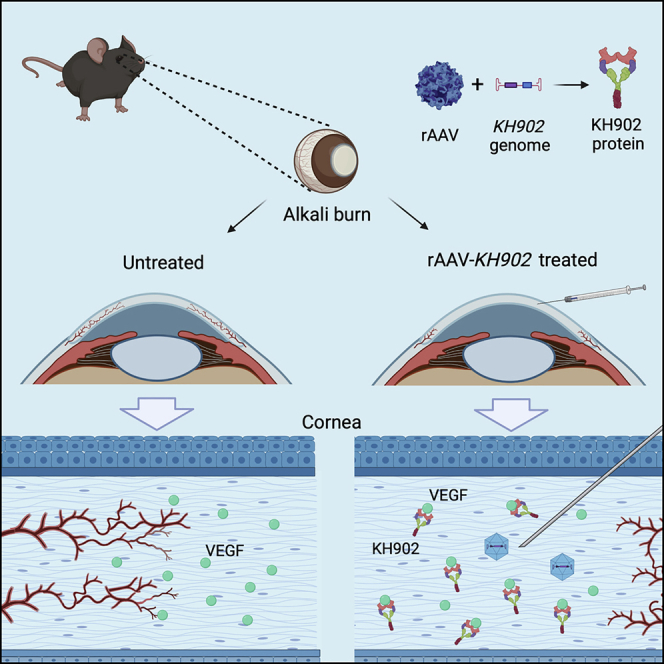

## Teaser

Currently, the most promising anti-VEGF therapies for corneal neovascularization (CoNV) are restricted by their short half-life and potential side effects of repeat dosing. Here, we engineered into AAV vectors the clinically proven anti-VEGF drug, conbercept (KH902), and demonstrated rAAV-*KH902* was efficacious and safe with long-term inhibition of CoNV following one dose.

## Introduction

The normal mammalian cornea is transparent and devoid of blood and lymphatic vessels. In pathological conditions where the balance of pro- and anti-angiogenic factors is disrupted, such as inflammation, hypoxia, and limbal barrier dysfunction, corneal neovascularization (CoNV) can occur. CoNV decreases corneal transparency and leads to visual impairment.^1^ It affects over 1.4 million people per year in the United States.^2^ Current treatments for CoNV include topical steroids and non-steroid anti-inflammatory administration, laser cauterization, fine needle diathermy, and amniotic membrane transplantation. However, all above methods have limited efficacies and come with related side effects.^1^ Vascular endothelial growth factor (VEGF) plays a critical role in corneal angiogenesis, but none of the aforementioned treatments specifically target this key molecule. The successful application of anti-VEGF agents in choroidal neovascularization (CNV)^3^ has led to a surge of interest in testing of these agents for their capacity to manage corneal angiogenesis in animal models and clinical trials. For instance, several early studies demonstrated the potential of bevacizumab in suppressing CoNV through topical, subconjunctival, perilimbal, and intrastromal administration in many animal models and clinical trials.^4^, ^5^, ^6^, ^7^, ^8^, ^9^ Since then, more anti-VEGF medications, such as ranibizumab and aflibercept, have been developed and applied in CoNV clinical therapy.^10^, ^11^, ^12^ However, because of their short half-lives, the limited durability of these VEGF-neutralizing proteins is an evident obstacle to achieve sustainable and efficacious treatment for CoNV.^13^, ^14^, ^15^ The remarkable advancement of gene-therapy technologies has inspired efforts to elevate the durability of anti-VEGF agents by packaging an expression cassette that encodes for a VEGF-neutralizing protein into recombinant adeno-associated virus (rAAV) vectors, which are highly attractive vehicles for the *in vivo* delivery of therapeutic transgenes in ocular diseases.^16^^,^^17^ rAAVs are favorable because of their low immunogenicity, genotoxicity, and high transduction profiles. A single dose of rAAV vector is capable of mediating robust and sustained gene expression,^18^^,^^19^ which is important for the goal of achieving therapy and mitigating the treatment burden for patients with chronic corneal diseases.

Conbercept, also known as KH902, is an anti-VEGF drug, which is a soluble recombinant protein made by fusing the second immunoglobulin (Ig) domain of VEGF receptor (VEGFR)1 and the third and fourth Ig domains of VEGFR2 with the Fc portion of IgG.^20^ Hereinafter, “conbercept” will refer to the complete manufactured drug, whereas “KH902” will refer to the recombinant transgene and its expressed products used in our study. Conbercept has been widely applied intravitreally in patients with exudative age-related macular degeneration, diabetic macular edema, retinal vein occlusion with macular edema, and retinopathy of prematurity with minimal or no adverse effects.^21^, ^22^, ^23^, ^24^ Several animal experiments have also demonstrated its sustainable efficacy in inhibiting CoNV following frequent doses of topical, intrastromal, or subconjunctival administration.^25^, ^26^, ^27^ In this study, we aim to develop a novel therapeutic approach using rAAV-mediated exogenous KH902 expression with a single dosing to steadily prevent and inhibit angiogenesis in the injured corneas.

## Results

### Intrastromal injection of rAAV2 and rAAV8 vectors produces efficient corneal cell transduction

**Figure 1:**
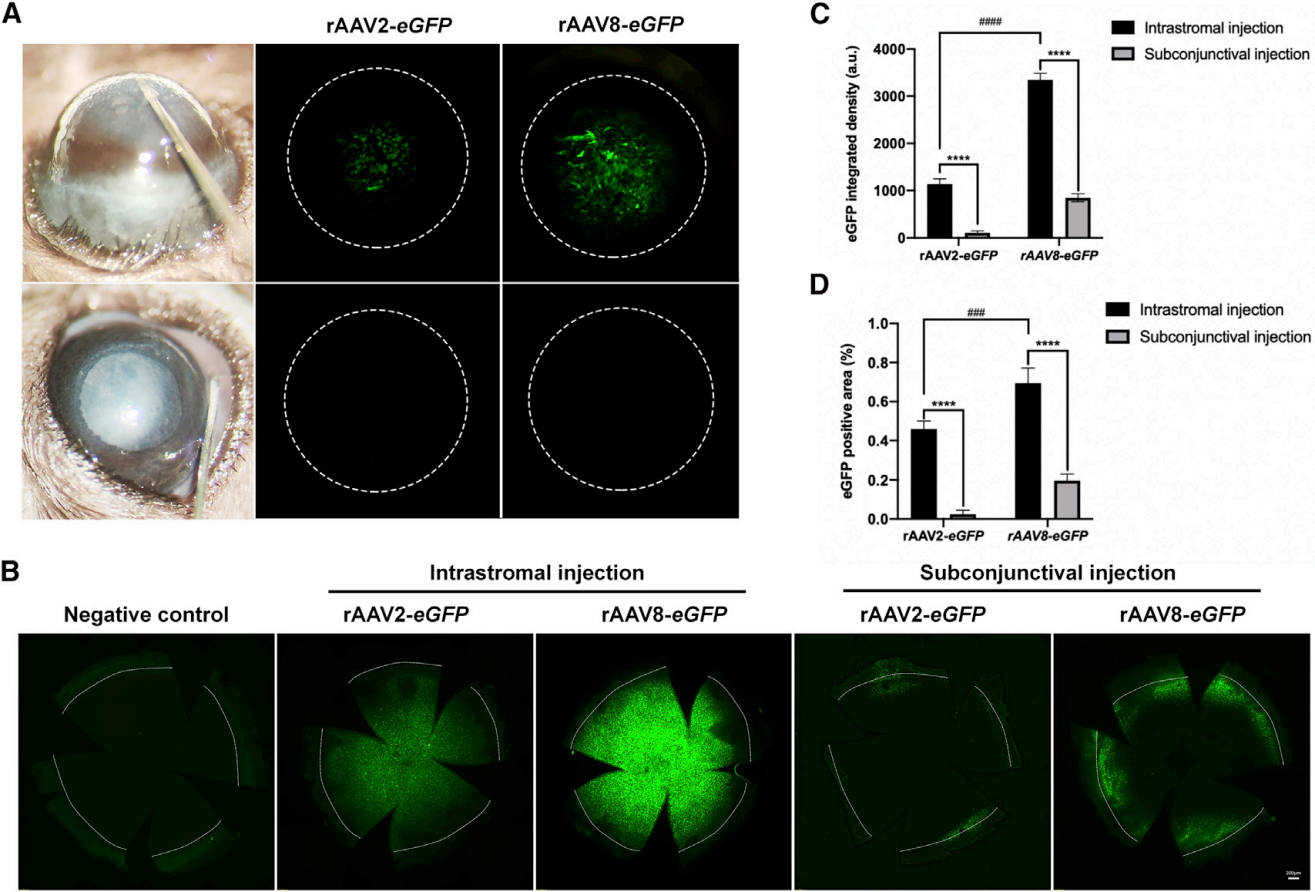
Comparison of corneal transduction between intrastromal and subconjunctival injections with rAAV2-*eGFP* and rAAV8-*eGFP* (A) Intrastromal injection (upper panel) and subconjunctival injection (lower panel) in the mouse cornea. The eGFP signal was detected by live animal imaging at 2 weeks post-intrastromal injection with rAAV2-*eGFP* (middle panel) and rAAV8-*eGFP* (right panel; 1.6 × 10^10^ genome copies [GCs] in 4 μL per cornea). The dotted circles represent the edge of mouse cornea. (B) Fluorescence microscopy of eGFP expression in representative corneal flat mounts in indicated groups from (A), respectively. The dotted lines represent the edge of mouse cornea. Scale bar, 200 μm. (C and D) Quantitative analysis of eGFP^+^ integrated density and area in cornea flat mounts in indicated groups from (B) (###p < 0.001, ∗∗∗∗p < 0.0001). Data are shown as mean ± SEM, n = 5 mice/group.

To ensure successful rAAV-mediated gene therapy, the route of application must allow efficient delivery and expression of the therapeutic gene inside the target tissue. Currently, therapeutic agents targeting the cornea are mainly administered via topical instillation, subconjunctival injection, or intrastromal injection. Topical instillation of rAAV vectors without the removal of the superficial epithelium have demonstrated relatively low transduction efficiencies;^28^ therefore, we primarily compared the biodistribution of rAAV vectors following subconjunctival and intrastromal injection routes. We injected mouse cornea with rAAV2 or rAAV8 packaged with the *eGFP* reporter transgene under a chicken β-actin ubiquitous promoter (rAAV2-*eGFP* or rAAV8-*eGFP*) at equal doses (1.6 × 10^10^ genome copies [GCs]/cornea), either via intrastromal or subconjunctival routes (Figure 1A). The level of eGFP expression in the cornea was assessed at 2 weeks post-administration through the direct detection of the eGFP signal by the live animal imaging system (Micron IV camera). Intriguingly, the eGFP signal was successfully detected in the corneas of mice treated by the intrastromal route but not by the subconjunctival route (Figure 1A). To further confirm the eGFP transduction pattern in the cornea, eGFP fluorescence was analyzed in corneal flat mounts at 2 weeks post-administration. Consistent with the live imaging data, eGFP was expressed in the entire cornea, including the limbus, with high integrated density following intrastromal injection of vector. In contrast, the expression was restricted around the limbus and peripheral cornea, with significantly weaker integrated density after subconjunctival injections. Moreover, rAAV8 has been shown to be more efficient than rAAV2 for transgene expression (Figures 1B−1D). Interestingly, we observed the eGFP signal within the extraocular muscle and sclera layer post-subconjunctival injection of rAAV8-*eGFP* compared to non-injected control (Figure S1). However, there was little to no detectable signal in the conjunctiva and optic nerve following subconjunctival injection of rAAV8. These results are consistent with the previous report.^29^ Together, these data demonstrated that intrastromal injection is a more effective route for administering rAAV2 and rAAV8 vectors for widespread transduction of the cornea.

### Corneal cell tropism and kinetics of rAAV2- and rAAV8-mediated eGFP and KH902 expression

**Figure 2:**
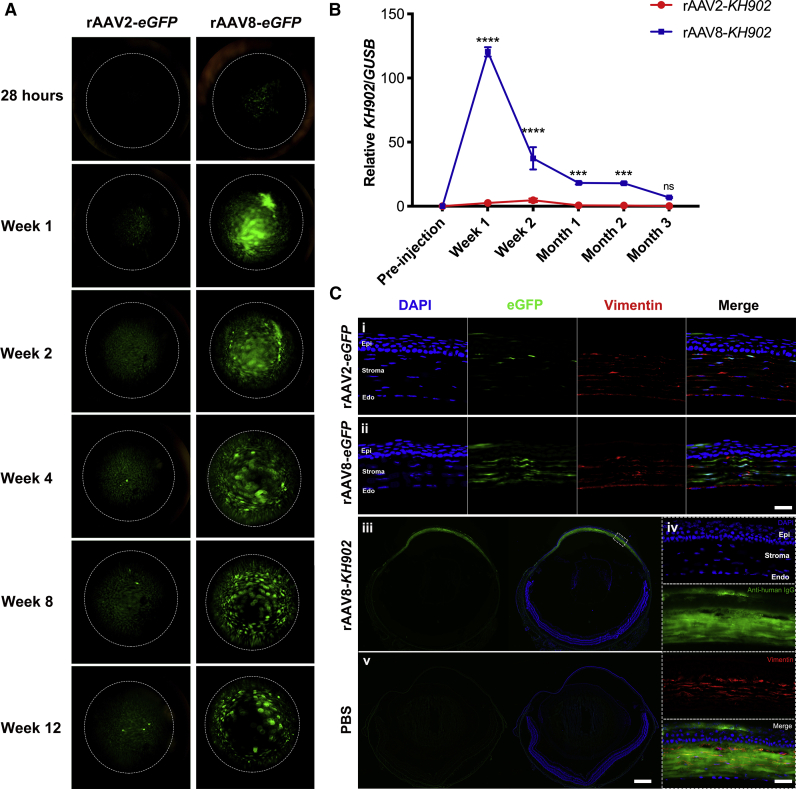
rAAV2- and rAAV8-mediated KH902 expression kinetics and cell tropism (A) rAAV2- and rAAV8-mediated eGFP expression detected at same intensity by live imaging microscopy at different time points, until 3 months (12 weeks) post-intrastromal injection. The dotted circles represent the edge of mouse cornea. (B) Relative *KH902* mRNA expression in rAAV8 and rAAV2 vector-treated mouse corneas (∗∗∗p < 0.001, ∗∗∗∗p < 0.0001). Data are shown as mean ± SEM, n = 4 mice/group. (C) Histological analysis of cell specificity in cornea sections with rAAV2 and rAAV8. (i and ii) Anti-vimentin staining (red) of mouse corneas at 2 weeks after intrastromal injection with rAAV2- and rAAV8-mediated eGFP expression. The eGFP signal in the corneal stroma co-localized with the vimentin-labeled keratocytes. (iii and v) Anti-human IgG (H+L) labeling KH902 protein (green) in the section of cornea intrastromally treated with rAAV8 or PBS, respectively. (iv) Higher magnification of the dashed-box regions in (iii) co-stained with anti-vimentin co-staining (red). (i, ii, and iv) Scale bars, 25 μm; (iii and v) scale bar, 500 μm. Epi, epithelial layer; Endo, endothelial layer. The dose of each rAAV vector for all of the above experiments was 1.6 × 10^10^ GCs in 4 μL PBS per cornea.

To inspect the kinetics of transgene expression mediated by rAAV2 and rAAV8 via corneal intrastromal injection, rAAV2-*eGFP* or rAAV8-*eGFP* was administered intrastromally at an equal dose of 1.6 × 10^10^ GCs per cornea. The eGFP signal mediated by rAAV8 was readily detected as early as 28 h post-injection by the live animal imaging. However, the eGFP signal in rAAV2-*eGFP*-treated corneas was not observed until 1 week post-administration, with a much weaker signal intensity than those conferred by rAAV8-*eGFP* treatment (Figure 2A). We next injected rAAV2-*KH902* or rAAV8-*KH902* intrastromally into wild-type mouse corneas at 1.6 × 10^10^ GCs per cornea and evaluated the relative *KH902* mRNA expression at weeks 1 and 2 and at months 1, 2, and 3 by droplet digital PCR (ddPCR). Robust expression of *KH902* mRNA was detected in the rAAV8-*KH902* group, reaching its peak at 1 week post-injection. Meanwhile, rAAV2 also led to detectable *KH902* expression but at significantly lower levels and with a lagging peak at 2 weeks post-injection. After reaching peak levels, *KH902* mRNA expression that was mediated by rAAV2 and rAAV8 gradually declined but remained detectable for up to 3 months post-injection, the final time point in our experiment (Figure 2B). Notably, relative *KH902* mRNA expression that was mediated by rAAV8 was higher than what was achieved by rAAV2 at every time point. This observation was consistent with eGFP signals conferred by the rAAV-*eGFP* vectors (Figure 2A).

To investigate the tropism of rAAV2 and rAAV8 transduction in the mouse cornea, corneal tissue sections from intrastromal injections with rAAV2-*eGFP* or rAAV8-*eGFP* were analyzed at 2 weeks post-injection. Results showed that the eGFP signal mainly co-localized with vimentin, a keratocyte marker, indicating that rAAV2 and rAAV8 mainly transduced keratocytes. Sporadic expression in epithelial cells was also observed. On the other hand, neither rAAV2-*eGFP* nor rAAV8-*eGFP* transduced corneal endothelial cells (Figure 2C, i and ii). Subsequently, we probed the distribution of KH902 protein using an anti-human IgG antibody in the corneas that were transduced with rAAV2-*KH902* or rAAV8-*KH902* (1.6 × 10^10^ GCs/cornea). KH902 protein following rAAV2 and rAAV8 transduction was primarily found in keratocytes and rarely in corneal epithelial cells (Figures 2C, iii and iv, and S3), which was consistent with the eGFP expression pattern following rAAV2-*eGFP* and rAAV8-*eGFP* transduction. However, our results clearly show that KH902 protein distributed not only in cell bodies of keratocytes but also diffused throughout the whole corneal stromal layer. This is attributed to the fact that KH902 contains a secretory signal peptide at the N terminus, thus facilitating secretion of the KH902 protein from keratocytes into the corneal stromal matrix.

Overall, the data indicated that rAAV8 mediated a stronger and earlier onset of KH902 expression in the cornea following intrastromal delivery than by rAAV2, suggesting that rAAV8-*KH902* was superior to rAAV2-*KH902* in the treatment of CoNV. Moreover, the expression of KH902 that was mediated by rAAV8 was sustained for up to 3 months and dispersed through the entire corneal stromal layer. This finding implicated a potential robust capacity for rAAV8-*KH902* to neutralize VEGF and consequently, to attenuate CoNV.

### Characteristics of immunological responses to rAAV2-*KH902* or rAAV8-*KH902* in the mouse cornea

**Figure 3:**
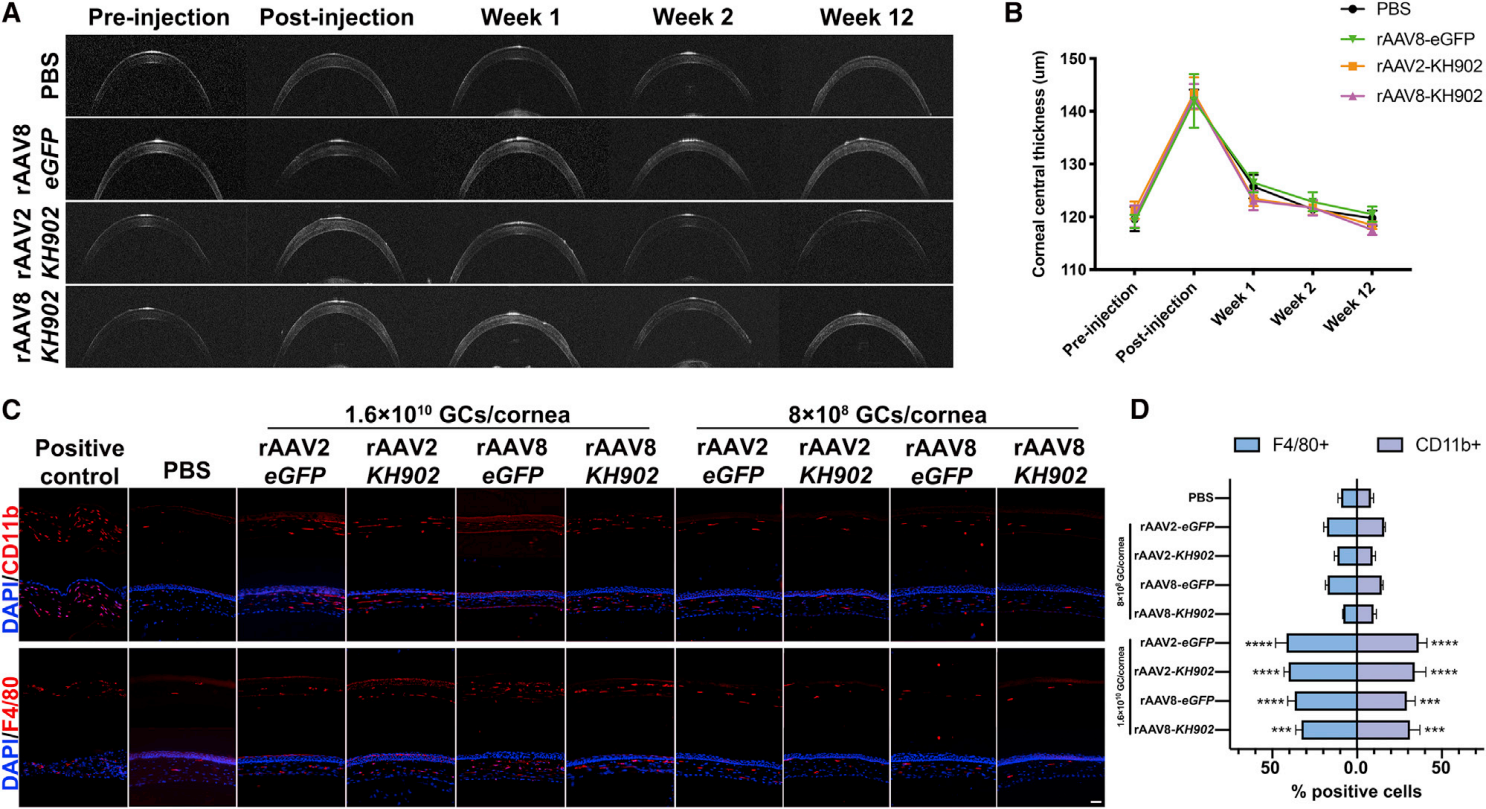
Central corneal thickness (CCT) and immune response toward rAAV2 and rAAV8 vectors (A) OCT images of corneas pre-injection, immediately post-injection and at weeks 1, 2, and 12 post-injection of PBS, rAAV2-*KH902*, and rAAV8-*eGFP*/*KH902* at the dose of 1.6 × 10^10^ GCs in 4 μL per cornea. (B) Quantitative analysis of CCT measured from (A) images. (C) Analysis of corneal immune responses to high (1.6 × 10^10^ GCs/cornea)- and low (8 × 10^8^ GCs/cornea)-dose rAAV2- or rAAV8-*eGFP*/*KH902* with immunofluorescent staining for monocytes/macrophages (CD11b, F4/80, red). Scale bar, 50 μm. (D) Calculated percentages of CD11b^+^ cells and F4/80^+^ cells in indicated groups from (C) data (∗∗∗p < 0.001, ∗∗∗∗p < 0.0001). Data are shown as mean ± SEM, n = 5 mice/group.

Given that the central corneal thickness (CCT) is an indicator of corneal health and physiological function,^30^ we analyzed CCT at various time points by optical coherence tomography (OCT) imaging following intrastromal injection with PBS, rAAV8-*eGFP*, rAAV2-*KH902*, and rAAV8-*KH902* (1.6 × 10^10^ GCs/cornea). Among the groups, PBS and rAAV8-*eGFP* were used as the injection control and the vector vehicle control, respectively. Immediately following the injection, we found the CCT was increased by 22.73 ± 2.93 μm in the PBS group, 23.50 ± 5.37 μm in the rAAV8-*eGFP* group, 22.12 ± 3.43 μm in the rAAV2-*KH902* group, and 22.75 ± 3.12 μm in the rAAV8-*KH902* group and then gradually reduced back to pre-injection levels at the end of week 12 (final time point of data collection) with normal morphological and anatomical structure under the OCT scan (Figures 3A and 3B). No significant difference of CCT was observed among all groups. All together, these data indicated that intrastromal injection with rAAV vectors and the *KH902* transgene product did not alter the CCT or disrupt the corneal physiological structure to produce local reactive corneal scarring or Descemet’s membrane detachment after 3 months. Meanwhile, we also assessed the immune response at 2 weeks post-injection of rAAV-*KH902* by estimating the level of monocyte/macrophage markers (CD11b, F4/80) in the cornea. The levels of CD11b^+^ or F4/80^+^ cells were significantly higher after high-dose (1.6 × 10^10^ GCs/cornea) rAAV2-*eGFP*/*KH902* and rAAV8-*eGFP*/*KH902* administration, whereas the percentages of CD11b^+^ or F4/80^+^ cells in their low-dose (8 × 10^8^ GCs/cornea) counterparts were significantly lower in comparison, which is at the similar levels as the PBS control (Figures 3C and 3D). To observe the early innate immune response in healthy corneas following low-dose rAAV vector administration, we analyzed and quantified CD11b-positive cells on day 4 after low-dose vector injection. The results showed that there was no significant difference among five injection groups with PBS, rAAV2-*eGFP*, rAAV8-*eGFP*, rAAV2-*KH902*, and rAAV8-*KH902*, but the immune response was much higher in all injection groups compared to the non-injected group (Figure S2). This result indicated that there is little to no early innate immune response induced by rAAV administration, and the immune response is mainly due to injection-related tissue damage. Therefore, the low-dose (8 × 10^8^ GCs/cornea) injection scheme for rAAV2-*KH902* and rAAV8-*KH902* delivery was used in subsequent *in vivo* CoNV therapy studies.

### Treatment with rAAV8-*KH902* via intrastromal administration effectively inhibits CoNV in an alkali-burn injury model

**Figure 4:**
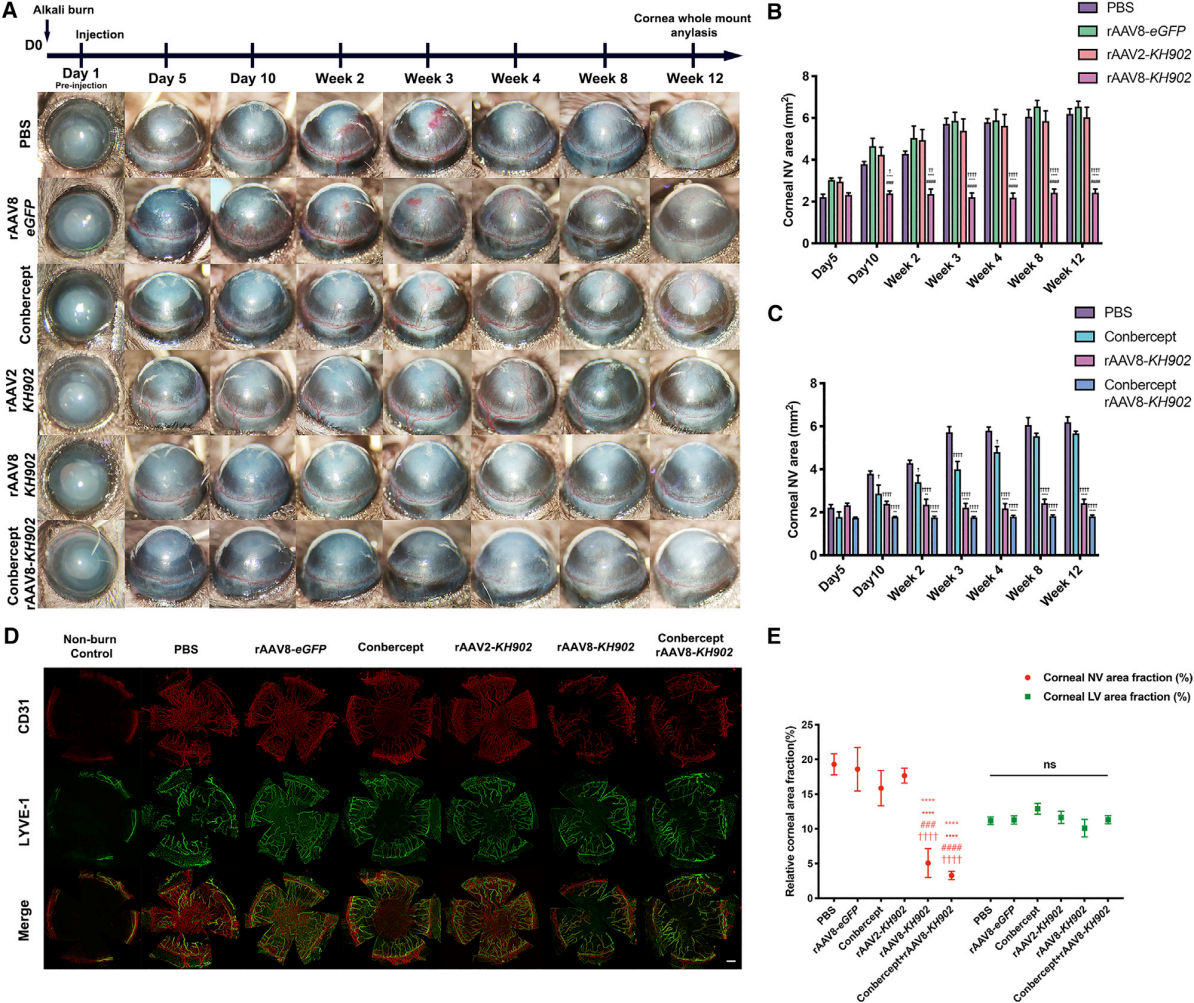
Long-term inhibition of CoNV by rAAV8-*KH902* via intrastromal delivery with the single dose in the alkali burn-induced CoNV model (A) Representative CoNV images of alkali-treated corneas injected with PBS, rAAV8-*eGFP*, conbercept (10 mg/mL, 4 μL), rAAV2-*KH902*, rAAV8-*KH902*, and rAAV8-*KH902* combined with conbercept (10 mg/mL, 4 μL) at days 5 and 10 and weeks 2, 3, 4, 8, and 12. (B and C) Histogram of CoNV area quantification in each condition from (A) data (†p < 0.05 significant difference from PBS group, ∗p < 0.05 significant difference from rAAV8-*eGFP* groups, #p < 0.05 significant difference from rAAV2-*KH902* group; ††, ∗∗, and ##p < 0.01; †††, ∗∗∗, and ###p < 0.001; ††††, ∗∗∗∗, and ####p < 0.0001). Data are shown as mean ± SEM, n = 5~7 mice/group. Two-way ANOVA and Tukey’s multiple comparison test were used. (D) Immunofluorescence analysis of mouse corneal flat mounts. The corneas in each condition of (A) harvested at 12 weeks after alkali burn were double stained, and the area covered by CD31^+++^/LYVE-1^–^ refers to blood vessels, and CD31^+^/LYVE-1^+++^ refers to lymph vessels (+++, strong positivity; ++, medium positivity; and +, mild positivity). Scale bar, 50 μm. (E) Corneal angiogenesis (neovascularization [NV]) and lymphangiogenesis (LV) analysis by measuring areas covered by CD31^+++^ and LYVE-1^+++^ staining, respectively, in each condition of (D) data (∗p < 0.05 significant difference from PBS group, •p < 0.05 significant difference from rAAV8-*eGFP* group, #p < 0.05 significant difference from conbercept group, †p < 0.05 significant difference from rAAV2-*KH902* group; ###p < 0.001; ††††, ∗∗∗∗, ••••, and ####p < 0.0001). Data are shown as mean ± SEM, n = 4 mice/group. The dose of each rAAV vector for the above experiments was 8 × 10^8^ GCs in 4 μL PBS or conbercept solution per cornea.

To test whether rAAV2-*KH902* and rAAV8-*KH902* by intrastromal delivery would therapeutically inhibit CoNV, we applied alkali burn on mice corneas to create the CoNV model and subsequently injected mice with PBS, rAAV8-*eGFP*, rAAV2-*KH902*, or rAAV8-*KH902* at the dose of 8 × 10^8^ GCs/cornea on day 1 post-alkali burn. We tracked CoNV progression at day 5, day 10, as well as 2, 3, 4, 8, and 12 weeks after corneal injury. At day 5 post-alkali injury, sprouting and splitting of the CoNV from limbal vascular plexus started to be visible in all groups, with no statistical differences among the groups in the CoNV area. Excitingly, the length and area of CoNV were not evidently increased in the rAAV8-*KH902* treatment group and remained relatively quiescent from day 5 to week 12 (experimental end point). On the contrary, rAAV2-*KH902* failed to inhibit the further growth of CoNV from the limbus to the central cornea at around 4 weeks post-burn, as it did in rAAV8-*eGFP* and PBS control groups (Figures 4A and 4B). To compare the therapeutic efficacy of rAAV8-*KH902* and the conbercept drug, we quantitively analyzed the area of CoNV in both of single-dose conbercept (10 mg/mL) and rAAV8-*KH902* treatment groups. The results showed that there was no significant difference at the early stages of CoNV growth at days 5 and 10. However, in the single-dose conbercept drug-treated group, CoNV burgeoned from 2 weeks post-treatment and further progressed to invade the central cornea at 4 weeks, ending up with intensive CoNV with sizes similar to the PBS control group at 12 weeks. The CoNV area size in the rAAV8-*KH902*-transduced group was significantly smaller compared to that in the conbercept drug-treated group from 2 weeks to 12 weeks post-injection, indicating that rAAV8-*KH902* exhibited prolonged anti-VEGF efficacy. In addition, rAAV8-*KH902* in combination with conbercept did not further inhibit the CoNV area compared to rAAV8-*KH902* alone throughout the observation period (Figures 4A and 4C), indicating at this dose, the expression of *KH902* delivered by rAAV8 was adequate to neutralize VEGF in a timely manner to achieve anti-angiogenic effects, and the use of conbercept as a supplement did not appear to be necessary to further strengthen the therapeutic effect. For verification, immunostaining data using anti-CD31 (also known as platelet-endothelial cell adhesion molecule 1 [PECAM-1]) for outlining the blood vessel on corneal whole mounts showed that the CoNV size correlated with the results of the gross vascular pathologies at 12 weeks (Figure 4D, upper panels).

Lymphangiogenesis has also been implicated in the pathological process of CoNV. The formation of new lymphatic vessels from the corneal limbus is believed to be similar to vascular growth during angiogenesis and is mainly induced by the binding of VEGF-C and VEGF-D to VEGFR2 and VEGFR3.^31^ Given that VEGF-A has also been shown to contribute to lymphangiogenesis,^31^ and conbercept blocks all VEGF-A isoforms, we next determined the effect of rAAV8-*KH902* on lymphangiogenesis. Since pathologic lymphatic vessels that invaded into cornea are not directly visible, we collected the mice corneas at week 12 in each group. Corneal whole mounts were double stained with CD31 as a pan-endothelial marker and LYVE-1 (lymphatic vessel endothelial receptor 1) as a specific lymphatic vessel marker. The area covered by CD31^+++^/LYVE-1^–^ blood vessels and CD31^+^/LYVE-1^+++^ lymph vessels^32^ was measured in cornea whole mounts. In all of the experimental groups at the 12-week follow-up, treatment with rAAV8-*KH902* alone and in combination with the conbercept drug remarkably reduced the size of angiogenesis, whereas there was no significant remission of lymphangiogenesis compared to the PBS control and various treatment groups (Figures 4D and 4E). Additionally, our data showed that rAAV8-*eGFP*/*KH902* did not induce lymphangiogenesis at long-term follow-up (Figure S4).

### Dll4/Notch signaling and extracellular signal-regulated kinase (ERK) activation are downregulated by rAAV8-*KH902*

**Figure 5:**
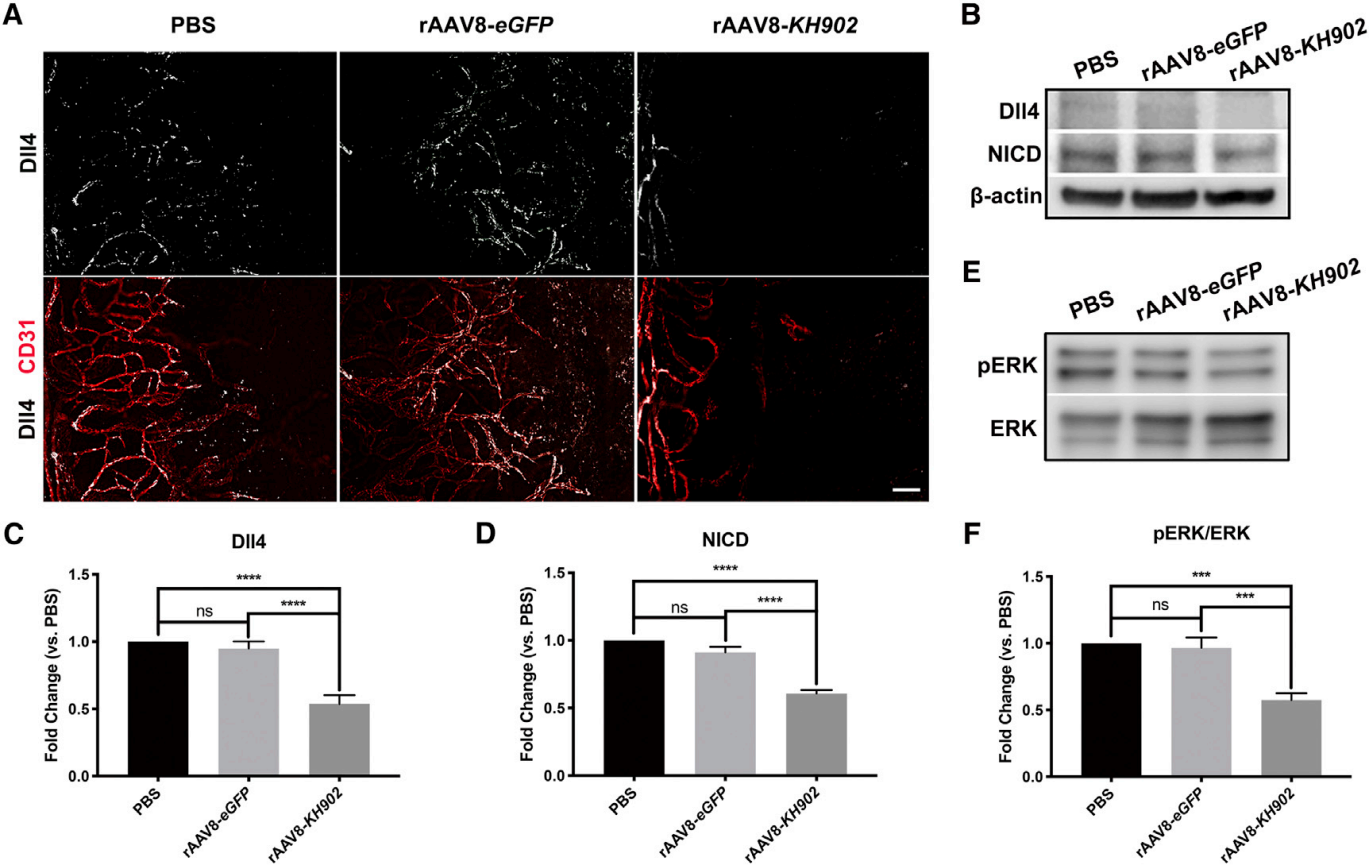
rAAV8-delivered KH902 downregulates Dll4/Notch signaling and ERK activation in the alkali burn-induced CoNV model (A) Immunofluorescence analysis of Dll4 expression in mouse corneal flat mounts co-stained with CD31 at 2 weeks post-alkali burn in PBS-, rAAV8-*eGFP*-, and rAAV8-*KH902* (8 × 10^8^ GCs/cornea)-treated corneas. Scale bar, 100 μm. (B−D) Western blot with semiquantitative analysis of Dll4 and NICD expression in mouse cornea at 2 weeks after alkali burn in each indicated treatment group (∗∗∗p < 0.001, ∗∗∗∗p < 0.0001). Data are shown as mean ± SEM, 6 corneas are pooled as an individual sample, and n = 3 samples/group. (E and F) Western blot with semiquantitative analysis of ERK activation. The results are presented as the ratio of phosphorylated ERK (pERK) to total ERK (pERK/ERK) in the indicated treatment groups at 8 days after alkali burn (∗∗∗p < 0.001, ∗∗∗∗p < 0.0001). Data are shown as mean ± SEM, 6 corneas are pooled as an individual sample, and n = 3 samples/group.

Sprouting angiogenesis is led by endothelial “tip” cells, directing the sprouting process, whereas endothelial “stalk” cells elongate the neovessel sprouts. During this process, VEGF and Notch signaling pathways are implicated in the selection of tip and stalk cells in the vascular endothelium. Specialized endothelial tip cells lead the outgrowth of blood-vessel sprouts toward the VEGF-A gradient.^33^ Dll4 and reporters of Notch signaling are distributed in a mosaic pattern among endothelial cells (ECs) of actively sprouting vessels.^34^ Under VEGF stimulation, quiescent endothelial cells are induced to form the tip cell filopodia and upregulate the level of Dll4 expression in the tip cells. In turn, the Dll4 ligand activates Notch signaling in the stalk cells, leading to the release of the active Notch intercellular domain (NICD) from the cell membrane, consequently enabling adequately spaced branching and sprouting.^34^ Therefore, we evaluated Dll4/Notch signaling expression in mouse cornea with vigorously growing vessels by immunostaining and western blot analyses at 2 weeks post-alkali burn. In PBS and rAAV8-*eGFP* control groups, Dll4 was broadly expressed in the corneal neovessel sprouts, suggesting an involvement of Dll4 in the process of corneal angiogenesis. By contrast, in the rAAV8-*KH902*-treated group, Dll4 was rarely detected, and the tip cell filopodia were completely retracted (Figure 5A). These results demonstrated that VEGF-A stimulation was blocked by rAAV8-*KH902*, thus preventing tip cell migration and CoNV progression. We also confirmed our findings by western blot results, which showed significant downregulation of Dll4 and NICD expression in the rAAV8-*KH902*-treated group compared to control groups (Figures 5B−5D).

VEGF binding to VEGFR results in phosphorylated VEGFR2, initiating downstream signaling pathways relevant to angiogenesis and producing several cellular responses in ECs.^35^ Among these pathways, VEGF-induced ERK1/2 signaling has been extensively studied and is shown to regulate microvascular endothelial differentiation and proliferation.^36^ We therefore collected mouse corneas at 8 days post-alkali burn to assess the level of ERK activation in each condition. The ratio of phosphorylated ERK (pERK) to total ERK (pERK/ERK) was significantly decreased in the rAAV8-*KH902*-treated group compared to the PBS group and the rAAV8-*eGFP*-treated group by western blot analysis (Figures 5E and 5F), suggesting that blocking VEGF by rAAV8-*KH902* resulted in the inhibition of ERK activation in alkali burn-induced CoNV mice.

### rAAV8-*KH902* prevents progression of pre-existing neovascularization in both alkali-burn- and suture-induced CoNV models

**Figure 6:**
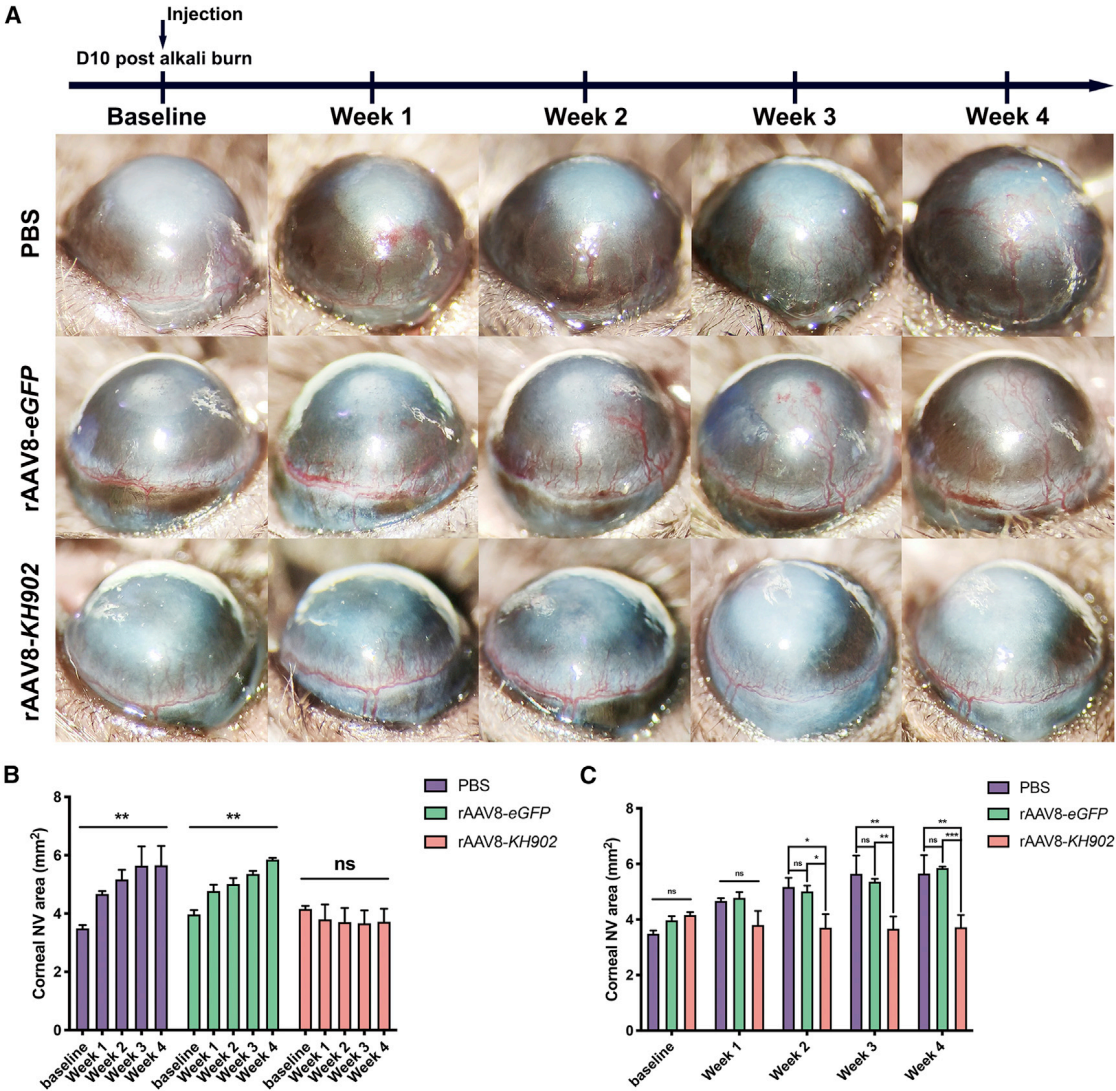
rAAV8-*KH902* prevents progression of pre-existing CoNV in the alkali-burn injury model (A) Mouse corneas were performed with intrastromal injection of PBS, rAAV8-*eGFP*, and rAAV8-*KH902* (8 × 10^8^ GCs in 4 μL per cornea) at day 10 after alkali burn (baseline). Representative images of CoNV observed weekly over 4 weeks are shown. (B) Quantification analysis of weekly CoNV area in each group shown in (A). The asterisk indicates significant differences between the end time point of observation (week 4) and corresponding baseline (∗∗p < 0.01). Data are shown as mean ± SEM, n = 5 mice/group. (C) Weekly parallel comparison by quantified CoNV area between experimental groups of (A) for 4 weeks (∗p < 0.05, ∗∗p < 0.01, ∗∗∗p < 0.001). Data are shown as mean ± SEM, n = 5 mice/group.

Chemical burn is an acute ocular injury and a complex condition with varied severity and offending lesions.^37^ For cases that fail to acquire immediate or intensive management at the initial stage, the CoNV is prone to progress rapidly during the active stage.^38^ It is thus of great interest to explore if rAAV8-*KH902* is capable of suppressing or even regressing the actively expanding CoNV triggered by alkali burn. Mouse corneas were injected intrastromally with PBS, rAAV8-*eGFP*, or rAAV8-*KH902* (8 × 10^8^ GCs/cornea) at 10 days after alkali burn, at which time, CoNV had already invaded into the cornea to varying degrees and continued growing (Figure 6A). Once CoNV induced by alkali burn was established, no remarkable regression was found in any of the experimental groups through the self-controlled study method (Figures 6A and 6B). However, in the rAAV8-*KH902* treatment group, the existing CoNV was significantly suppressed and maintained at the pre-treatment state during the 4-week follow-up (Figures 6A and 6C).

**Figure 7:**
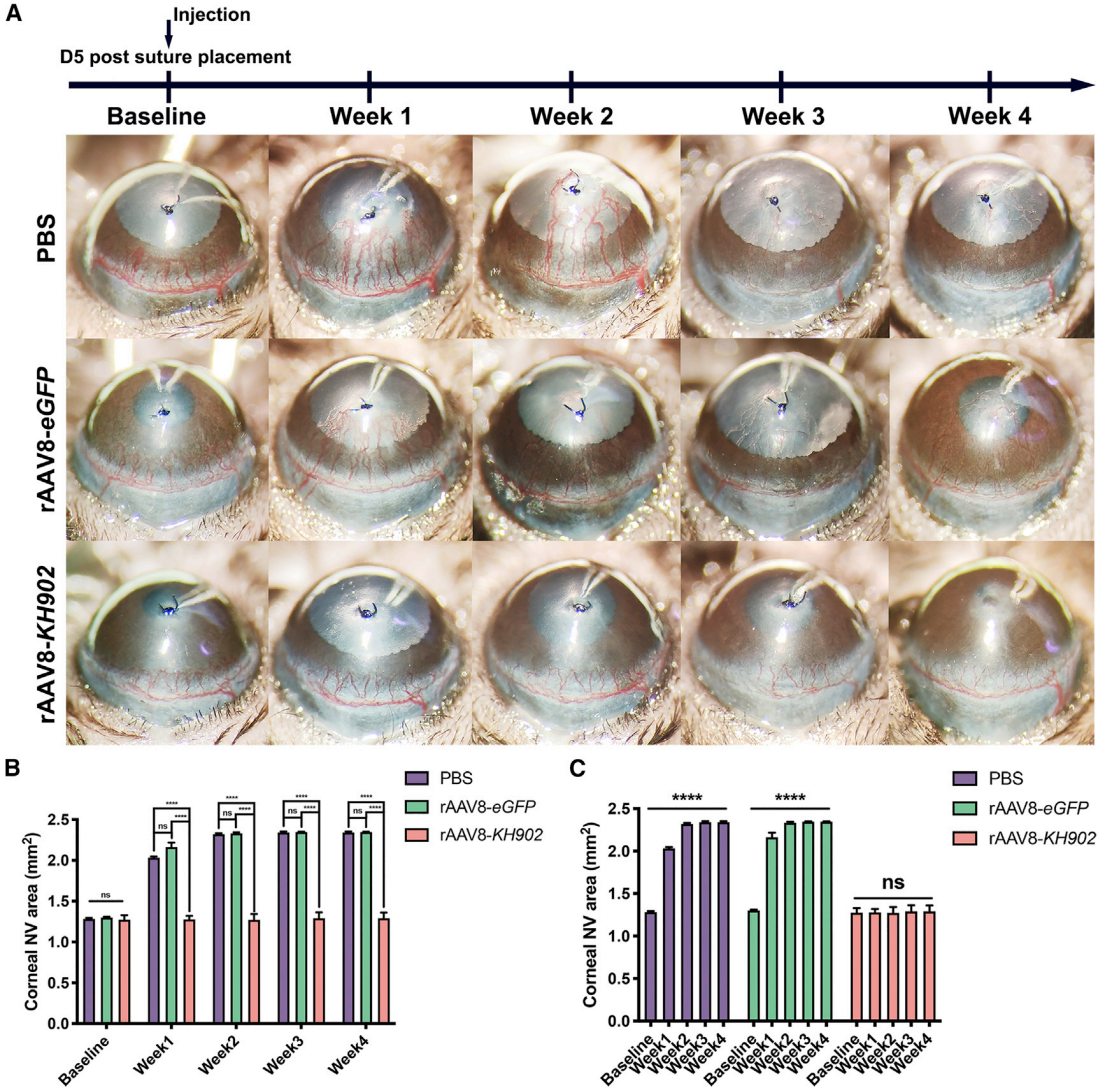
rAAV8-*KH902* prevents progression of pre-existing neovascularization in the suture-induced CoNV model (A) Mouse corneas subjected to 5-day suture placement (baseline) were treated with intrastromal injection of PBS, rAAV8-*eGFP*, and rAAV8-*KH902* (8 × 10^8^ GCs in 4 μL per cornea). Weekly representative images of CoNV are shown with a 4-week follow-up. (B) Quantification analysis of CoNV areas with comparing experimental groups from (A) for 4 weeks (∗∗∗∗p < 0.0001). Data are shown as mean ± SEM, n = 4~7 mice/group. (C) Quantification analysis of weekly CoNV areas in each group from (A) data. The asterisks indicate significant differences between the end time point (week 4) and the baseline (∗∗∗∗p < 0.0001). Data are shown as mean ± SEM, n = 4~7 mice/group.

To confirm the effect of rAAV8-*KH902* at inhibiting CoNV in a different injury model, we further explored it using the suture-induced CoNV mouse model by placing an intrastromal suture with a knot on the mouse cornea. After 5 days of suture stimulation, CoNV was actively growing and had invaded into the cornea. At this time, mice were injected with PBS, rAAV8-*eGFP*, or rAAV8-*KH902* intrastromally at a dose of 8 × 10^8^ GCs per cornea. The level of CoNV progression was tracked and quantified before and after injection. Compared with the PBS and the rAAV8-*eGFP* controls, the progression of CoNV was significantly inhibited with rAAV8-*KH902* treatment, and the inhibitory effect was sustained to our final time point (Figures 7A and 7B). Nevertheless, no regression of the established CoNV was observed following rAAV8-*KH902* treatment (Figures 7A and 7C). Therefore, our data confirmed that rAAV8-*KH902* had sustained a therapeutic effect on existent CoNV in the active stage.

## Discussion

CoNV severely affects visual function and can be a pathological sequel of multiple etiologies, such as contact lens wear; dry eye; trauma; chemical burn; limbal stem cell deficiency; ocular surface inflammation; and corneal infections with bacteria, fungus, and virus.^1^^,^^2^ Current therapies are limited by efficacy and safety concerns.^2^ Intrastromal injection of conbercept can inhibit CoNV, but it requires repeated dosing and produces injection-associated side-effects.^39^ AAV-based gene therapy for CoNV has the potential to overcome these issues by ideally providing a sustained supply of anti-VEGF protein at the site of the disease following a single vector treatment. Toward this end, we explored the use of rAAV vectors to mediate KH902 expression to treat CoNV. Currently, there are several anti-VEGF agents mainly used in the ophthalmology clinic to treat retinopathies, including bevacizumab, ranibizumab, aflibercept, and conbercept. All of these agents have high binding affinity to VEGF, and they block the VEGF signaling pathway with high efficiency.^40^ However, up to now, only one product (aflibercept) has been vectorized^41^^,^^42^ and tested in clinical trials for retinal vasculopathy (ClinicalTrials.gov: NCT04418427 and NCT04645212), and none of them have been reported to treat corneal diseases as a vectored transgene for therapy. Our report represents the earliest study to engineer into an AAV vector the clinically proven anti-VEGF drug, conbercept, to treat CoNV. More importantly, we demonstrate that our vector construct (rAAV8-*KH902*) is efficacious and safe and confers stable inhibition of CoNV following one dose. We believe that our efforts in this study will provide strong support for the future translation of AAV-based gene therapy of CoNV. Indeed, many other pre-clinical studies have demonstrated that rAAV-based therapies can treat CoNV through the delivery of various transgenes that interfere with angiogenesis, such as VEGFR1 (sFlt-1), endostatin, angiostatin, decorin, human leukocyte antigen G (HLA-G), microRNAs, etc.^43^, ^44^, ^45^, ^46^, ^47^, ^48^ However, aside from anti-VEGF protein drugs aforementioned, none of these candidates have been approved for clinical application. In comparison, conbercept (KH902) has been used in patients with ocular diseases. Additionally, since angiogenesis sustains inflammation by providing the cells with oxygen and nutrients for the metabolic needs at inflammatory sites,^49^ rAAV8-*KH902* also decreased some degree of inflammation under alkali burn and suture-induced injury models in our study as a result of its ability to inhibit angiogenesis (Figure S5).

AAV serotype tropism and the route of administration play pivotal roles in the pattern of AAV vector biodistribution and transgene expression.^50^ Various routes of administration are being explored for AAV gene delivery to the cornea in larger animals, such as topical, subconjunctival, and intrastromal delivery routes. Topical application, the easiest route of administration, has a relatively low transduction efficiency, and the transduction of non-target tissues when vector is spread through tears remains a potential adverse effect.^28^ Subconjunctival injection is relative easy and simple, leading to the potential transduction of a wide range of ocular tissues.^29^ Intrastromal injection, technically difficult for beginners, has demonstrated serotype-dependent transduction of different anterior eye segments.^50^ However, there is no evidence to compare the transduction efficiency of AAV vectors in the cornea between subconjunctival and intrastromal delivery routes. According to our data, they revealed that intrastromal delivery of rAAV2 or rAAV8 vectors generated more efficient and widespread transduction of the cornea than achievable by subconjunctival injection at the same dose. Our result also showed that subconjunctival injection of rAAV vectors led to robust transduction in periocular tissues, such as extraocular muscles (Figure S1), consistent with previous studies.^29^^,^^51^ Interestingly, subtle differences such as injection volume and dose can influence the vector distribution. For instance, it was recently demonstrated that dogs receiving the highest dose and volume of AAV8G9 vectors, generated neutralizing antibodies (NAbs) against the capsid. This outcome was consistent with the hypothesis that a threshold amount of AAV vectors in systemic circulation must be reached to produce a capsid antibody response.^52^ In our study, a 4-μL intrastromal injection volume was used to allow the agents to spread completely throughout the entire cornea; however, this might have resulted in some tissue transduction beyond the cornea. In addition, an AAV8 capsid NAb response was detected in all cases at 2 weeks post-AAV8 vector administration under alkali injury (Table S1). This result may be related to the administrated volume and/or perhaps to the severe response under corneal trauma. Above all, the volume and dose of AAV vectors should be prudently considered in the translation of gene therapy for corneal diseases in large animals and clinical trials.

In our study, we found that rAAV2- and rAAV8-mediated transgene expression in the mouse cornea reaches a peak at first and then decreases gradually to a lower level where expression maintains stable for a relatively long time. Recently, a few clinical gene-therapy studies have raised concerns about long-term outcomes of rAAV-mediated gene transfer.^53^ Two clinical trials in patients with hemophilia who received rAAVs packaging factor IX or factor VIII found that the levels of these factors were stable during the first year but gradually declined after that.^54^^,^^55^ The factors causing the later decline in expression are not entirely known. We suspect several possible reasons for the declining corneal transgene expression observed in our study. (1) Intrastromal injection results in the transient separation of the stromal matrix, allowing the injection fluid to be distributed throughout the cornea. The cornea would then return to the pre-injection state.^56^ This process may activate corneal repair mechanisms, as keratocytes immediately adjacent to the lesion begin to undergo apoptosis, and neighboring keratocytes begin to divide and migrate toward the damaged area. Dilution of the episomal vector genomes due to cell division and the clearance of apoptotic transduced cells would be challenges in achieving long-term gene expression.^56^ (2) A portion of corneal epithelial cells were also transduced after rAAV vectors were injected, likely due to the leaking into the epithelium alongside the needle’s trajectory. Corneal epithelium is a self-renewing tissue and regenerates every 1–2 weeks.^57^ As epithelial cells renew, rAAV-mediated gene expression is lost. (3) Numerous rodent studies have demonstrated that most AAV vector-encoded transgenes are immunologically inert after delivery.^58^ However, a study from Wang et al.^59^ demonstrated that a local immune response limits sustained expression of a secreted protein in muscle gene transfer. Breous and colleagues^58^ found that the presence of inflammation influenced the stability of AAV transgene expression in the mouse liver. Our kinetics study was performed with high-dose vectors, in which corneal immune response was strongly induced following rAAV injection. Thus, the involvement of rAAV-mediated inflammation should not be excluded.

Angiogenesis is the formation of new vessels from pre-existing blood vessels. It is not only dependent on endothelial cell (EC) proliferation and invasion but also requires subsequent pericyte coverage for vascular stabilization and maturation.^60^ In the absence of pericytes, newly formed ECs are unstable and prone to regression without VEGF stimulation, suggesting immature vessels depend on VEGF for survival and growing.^61^ Multiple studies on anti-VEGF monotherapy showed that mature vessels did not regress, since these vessels depend less on VEGF.^62^ Our observations are compatible with these previous findings. Therefore, the earlier rAAV8-*KH902* treatment for CoNV would achieve better outcomes.

Lymphangiogenesis is usually concurrent with hemangiogenesis in the human cornea. Our results showed that rAAV8-*KH902* conferred significant anti-angiogenesis effects, whereas lacking marked inhibition of lymph vessels following alkali burn-induced CoNV. KH902 has a very high affinity for all VEGF-A isoforms, placental growth factor 1 and 2 (PlGF1 and -2) and VEGF-B, but not high for VEGF-C and VEGF-D.^40^ VEGF-C, VEGF-D, and their cognate receptor VEGFR3 are main mediators of lymphangiogenesis, whereas VEGF-A only has partial involvement.^31^ Therefore, KH902 presumably has limited inhibitory effects on lymphangiogenesis. This speculation is consistent with a previous study that tested aflibercept for treating a high-risk corneal transplantation model. Hemangiogenesis was shown to be strongly inhibited, whereas lymphangiogenesis was only slightly blocked; however, anti-VEGF-C and soluble VEGFR3 efficiently reduced lymphangiogenesis.^63^ Furthermore, not only was the VEGF family able to modulate lymphangiogenesis during alkali burn injury, but also other growth factors and cytokines, such as insulin-like growth factors (IGFs), hepatocyte growth factor (HGF), fibroblast growth factors (FGFs), and interleukins (ILs), also played key roles.^64^ In addition, although the lower doses used were sufficient to neutralize VEGF-A/B, PlGF, and effectively reduced angiogenesis, they may not be sufficient to achieve significant lymph vascular relief. Thus, the tolerable dose of rAAV-mediated KH902 to achieve the best therapeutic effect is also a focus of future research.

The window of anti-angiogenic treatment of CoNV is difficult to determine, since different cases have distinct pathological etiologies. The pattern of angiogenesis and the proper therapeutic course strongly depend on the characteristics of preceding stimuli and the underlying pathologies. For instance, in herpes simplex virus (HSV)-induced keratitis, CoNV can be evident as early as day 1 and may continue to up to 3 weeks after corneal HSV-1 infection. As the disease progresses, infection, inflammation, and CoNV will trigger each other in a positive feedback loop, leading to an extended course.^65^ In other cases, patients with severe chemical injuries could enter a chronic phase that may persist for more than 6 weeks, developing significant limbal stem cell deficiency and complications with neovascularization.^37^ Our data showed that a single dose of rAAV8-*KH902* delivery offered at least a 3-month therapeutic window, whereas direct conbercept application can only last for 10−14 days. Therefore, rAAV8-*KH902* continually confers an anti-VEGF effect that significantly prolongs the therapeutic window. This makes a significant difference in reducing the need for repeated dosing of an anti-VEGF drug in patients with chronic corneal diseases.

In summary, rAAV8-*KH902* provides sustained therapeutic levels of anti-VEGF activity following a single intrastromal injection, leading to efficacious inhibition of CoNV for an extended period of time. This study demonstrates the potential long-acting and relative safety of rAAV-based, anti-VEGF gene therapy for CoNV.

## Materials and methods

### Vector production

The vectors were packaged with transgene cassettes encoding *eGFP* or *KH902* under the control of a chicken β-actin/cytomegalovirus (CMV) promoter. The vector encoding *KH902* was designed with a rabbit globin poly(A). Vectors were produced using triple transfection as described.^66^ Vectors were purified by CsCl gradient ultracentrifugation and titered by both ddPCR and silver staining of sodium dodecyl sulfate (SDS)-polyacrylamide gels.

### Animals

C57BL/6J mice were obtained from Jackson Laboratory (Bar Harbor, ME, USA), bred, and maintained in standardized conditions with a 12-h light/12-h dark cycle in the Animal Facility at the University of Massachusetts Medical School. All experiments were approved by the Institutional Animal Care and Use Committees and in line with Association for Research in Vision and Ophthalmology (ARVO)’s statement regarding the use of animals in ophthalmology and vision research.

### Alkali-burn-induced CoNV

The mouse model for alkali burn-induced CoNV was performed as previously described,^25^ with some modifications. Mice were anesthetized via an intraperitoneal injection of a ketamine (5 mg/mL) and xylazine (2 mg/mL) combination (10 mL/kg body weight), and the topical anesthetic proparacaine (0.5%) was applied on the corneal surface. Circular filter-paper discs (2 mm diameter) were pre-soaked in 1 M NaOH for 20 s and then placed on the center of the cornea of the right eye for approximately 40 s, followed by washing generously with 15 mL sterile saline solution for 1 min.

### Suture-induced CoNV

A modification from a previously described suture technique was performed to induce CoNV.^67^ Briefly, the right eye of each mouse received corneal suture placement under general anesthesia (an intraperitoneal ketamine [5 mg/mL] and xylazine [2 mg/mL] combination [10 mL/kg body weight]), supplemented by topical anesthesia (0.5% proparacaine). A single 10-0 nylon suture was placed intrastromally with a knot in the temporal cornea of the eye at 1 mm away from the limbus. To ensure the consistency and reproducibility of the procedure, the whole process was performed on each animal by the same researcher under a dissecting microscope.

### rAAV vector delivery by intrastromal or subconjunctival injection

Intrastromal injections were performed using a previously published method.^7^ In brief, an incision around 1.0 mm in size was first made in the corneal epithelium equidistance between the temporal limbus and the center of the cornea with the tip of a 30-gauge needle. Then 1.6 × 10^10^ or 8 × 10^8^ GCs of rAAV vectors in 4 μL of PBS were injected through the incision into the corneal stroma by using a 5-μL Hamilton syringe with a 34-gauge needle (Hamilton, Reno, NV, USA; 30° bevel angle). Subconjunctival injection was also performed by using a 5-μL Hamilton syringe. A total of 1.6 × 10^10^ GCs of rAAV vectors were injected into the upper, lower, nasal, and temporal subconjunctiva, respectively, with 1 μL (0.4 × 10^10^ GCs) injection per each site. Antibiotic ointment was applied after the injections.

### *In vivo* fluorescence imaging

Animals in each group were observed weekly for a total of 12 weeks after rAAV administration. eGFP expression in the mouse eye was captured by a Micron IV camera (Phoenix Research Labs, Pleasanton, CA, USA).

### Corneal OCT

Mouse corneal OCT was performed using a Micron IV OCT Imaging system (Phoenix Research Labs, Pleasanton, CA, USA). Mice were anesthetized via an intraperitoneal injection as aforementioned. The mouse cornea was placed toward the lens of the camera, and the entire anterior chamber was imaged. CCT was then measured in captured images by ImageJ software (available online at https://imagej.nih.gov/ij/). CCTs were measured before injection; immediately after injection; and at 1, 2, and 12 weeks post-injection.

### Anterior color imaging and quantification analysis of corneal neovascularization

Procedures were performed under general anesthesia and topical eyedrops as mentioned before. The mouse eye was placed under the ophthalmic surgical microscope (Wild Heerbrugg), and the cornea from different angles was imaged by a digital camera attached to the microscope. CoNV was analyzed at the set time points using ImageJ software. The area of CoNV was calculated by using the following formula: area (mm^2^) = CN/12 × 3.1416 × (R^2^ − [R − VL]^2^), where CN is the clock hours of neovascularization; R is the radius of the cornea; and VL is the longest vessel length, extending from the limbal vasculature.^44^ Color images of each cornea in a live mouse were performed in eight different angles, and the area of corneal neovascularization was calculated four times at each angle accordingly.

### Immunohistochemistry of whole-cornea flat mounts

Eyeballs were enucleated and fixed with 4% paraformaldehyde (PFA) for 1 h at room temperature after a small hole was made at the limbus with a needle. The excised eyeballs were then prepared for whole-mount staining with a modification to previous reports.^32^ In brief, the cornea and sclera were separated by the incision along the limbus, followed by removal of the lens and iris. Four radial cuts in the cornea were made to allow whole-mount flattening. To observe the eGFP expression, the eGFP signal and images were directly recorded using fluorescein isothiocyanate (FITC; 488 nm) with the Leica DM6 microscope with a 16-bit monochrome camera. For analysis of corneal angiogenesis and lymphangiogenesis, the tissues were washed by 0.3% Triton X-100 in PBS and blocked with blocking buffer (0.3% Triton X-100/5% normal bovine serum albumin [BSA]; Cell Signaling Technology)/1× PBS for 1 h. The corneas were stained overnight at 4°C with rat anti-CD31 (PECAM-1, 1:400, sc-18916; Santa Cruz, Santa Cruz, CA, USA), rabbit anti-mouse LYVE-1 (1:200, 11-034; AngioBio), goat anti-mouse Dll4 (1:40, AF1389; R&D Systems), or rat anti-mouse CD11b (1:50, #550282; BD Pharmingen). The primary antibodies were then detected with goat anti-rabbit, anti-rat, or donkey anti-goat secondary antibodies conjugated with Alexa Flour 488, 594, or 647 (Thermo Fisher Scientific, Singapore). After the final wash with 0.3% Triton X-100 in PBS, the corneal tissues were mounted endothelial side down and imaged by a Leica DM6 microscope with a 16-bit monochrome camera. Image processing was performed with Adobe Photoshop CC 2019 to improve definition. The eGFP fluorescent-integrated density (the sum of the values of the pixels in the image) and eGFP-positive area were analyzed with ImageJ software, and areas covered by blood, lymphatic markers, or CD11b^+^ cells were measured. Entire corneas were analyzed by two independent observers, blind to treatment status to minimize sampling bias.

### Histology and immunohistochemistry of cornea cryosections

The freshly excised eyeballs were directly embedded in optimal cutting temperature (OCT) compound (Fisher Scientific, Pittsburgh, PA, USA) in preparation for sectioning. 14 μm-thick cryosections were made from frozen blocks (Leica CM3050 S; Leica Biosystems, Buffalo Grove, IL, USA). Following the fixation of sections with 4% PFA for 15 min at room temperature, tissue sections were rinsed by 0.3% Triton X-100 in PBS and blocked with blocking buffer (1× PBS/1% BSA/0.3% Triton X-100) for 1 h. Slides were stained overnight at 4°C with primary antibodies. The primary antibodies used were rat anti-F4/80 (1:400, NB600-404; Novus), rat anti-mouse CD11b (1:50, #550282; BD Pharmingen), rabbit anti-vimentin (1:100, #5741; Cell Signaling Technology), and donkey anti-human IgG (H+L) conjugated with Alexa Fluor 488 that binds to the heavy (H) and light (L) chains of humanized IgG (1:400, #144222; Jackson ImmunoResearch Laboratories), which were all diluted in PBS with 0.3% Triton X-100 and 5% BSA. The secondary antibodies with 4′,6-diamidino-2-phenylindole (DAPI; #9542; Sigma-Aldrich) counterstain used were goat anti-rat IgG-Alexa Fluor 594 and goat anti-rabbit IgG-Alexa Fluor 594. Fluorescence images were acquired by a Leica DM6 microscope. Image analysis was performed with Adobe Photoshop software. CD11b^+^ or F4/80^+^ cells were detected and counted by using ImageJ software.

### *KH902* mRNA expression analyses

RNA from normal mouse corneas, treated or untreated with rAAV2-*KH902* or rAAV8-*KH902* (4 corneas/group), were isolated at weeks 1 and 2 and months 1, 2, and 3 post-treatment using the RNeasy Plus Micro Kit and reverse transcribed into cDNA using the QuantiTect Reverse Transcription Kit (both from QIAGEN, Hilden, Germany). Multiplexed ddPCR was performed using a QX200 ddPCR system (Bio-Rad Laboratories, Hercules, CA, USA) with probes targeting KH902 and the reference transcript, glucuronidase beta (*GUSB*; #4448489; Thermo Fisher Scientific). Primer and probe sets for *KH902* were designed and synthesized by Integrated DNA Technologies (Coralville, IA, USA) (forward: 5′-GGACATACACAACCAGAGAGAC-3′ and reverse: 5′-GTGAGTGAAAGAGACACAGGAA-3 and probe: 5′-/56-FAM/CCCATTTCA/ZEN/AAGGAGAAGCAGAGCCA/3IABkfq/-3′). *KH902* mRNA copy number was normalized to *GUSB* copies. The ddPCR results are presented as the ratio of *KH902* values to *GUSB* values.

### Western blot

Six corneas from each experimental group were combined, and total protein from pooled corneas in each group was extracted on ice in radioimmunoprecipitation assay (RIPA) lysis buffer with fresh protease and phosphatase inhibitors (Thermo Fisher Scientific, Waltham, MA, USA), following homogenization using QIAGEN TissueLysis II. A total of 20 μg/lane protein extract was loaded onto a 4%–12% Bis-Tris Precast Gel (QP3510; SMOBIO) and transferred onto polyvinylidene difluoride (PVDF) membranes (Millipore). Nonspecific binding was blocked with 5% BSA in Tris-buffered saline with Tween-20 (TBST). Membranes were incubated with rabbit anti-cleaved Notch1 (#4147; Cell Signaling Technology), goat anti-mouse Dll4 (AF1389; R&D Systems), rabbit anti-pERK1/2 (#4370; Cell Signaling Technology), and anti-ERK1/2 (#9102; Cell Signaling Technology) antibodies overnight at 4°C. Membranes were incubated with rabbit anti-ERK following membrane harsh stripping. After washing with TBST, the membranes were incubated with horseradish peroxidase-conjugated goat anti-rabbit IgG (1:10,000, G-21234; Invitrogen) or rabbit anti-goat IgG (1:1000; HAF017; R&D Systems) for 1½ h. Protein detection was performed using the Enhanced Chemiluminescence (ECL) Western Blotting Substrate (#W1001; Promega, Madison, WI, USA) in combination with the Odyssey system. The intensity of the specific bands was quantified using ImageJ software. The data represent three western blot experiments.

### NAb assay

The presence of NAbs in mouse sera was assayed following previously described methods with slight modifications.^68^ Briefly, mouse serum samples were collected 2 weeks after rAAV8-*KH902* injection and heat inactivated at 56°C for 35 min. AAV8.*CMV.LacZ* vectors (10^9^ GC/well) were incubated with 2-fold serial dilutions (initial dilution, 1:5) of heat-inactivated serum samples in DMEM for 1 h at 37°C. Subsequently, the serum-vector mixture was added to 96-well plates seeded with Huh7 cells (1 × 10^5^ cells/well) that had been infected with wild-type human adenovirus serotype 5 (HAdV5) for 2 h (50 viral particles/cell). After 1 h, each well was supplemented with 20% FBS in DMEM and incubated overnight at 37°C and 5% CO_2_. Cells were then lysed, and lysates were assayed with the mammalian β-galactosidase (β-gal) kit for bioluminescence (Applied Biosystems) and measured with a microplate luminometer (Clarity; BioTek). The NAb titer was reported as the highest serum dilution that inhibited AAV.*CMV.LacZ* transduction (β-gal expression) by ≥50% of naive mouse serum.

### Statistics

Results are expressed as mean ± SEM. Each data point represents the mean of 3 replicate values. Analysis was performed using one-way or two-way ANOVA for multiple variables, and Tukey’s multiple comparison test was used for inter-group differences using GraphPad Prism 7.0 (GraphPad Software, La Jolla, CA, USA). p < 0.05 was considered significant.

## Supplemental information

Document S1. Figures S1–S5 and Table S1

Document S2. Article plus supplemental information
