# Supplementary material for: Efficacious, safe, and stable inhibition of corneal neovascularization by AAV-vectored anti-VEGF therapeutics
Source: Mol Ther Methods Clin Dev. 2021 Jun 24;22:107–21. doi: 10.1016/j.omtm.2021.06.007 (PMC8413663; doi:10.1016/j.omtm.2021.06.007)
Supplement: Document S1. Figures S1–S5 and Table S1 [file mmc1.pdf]

OMTM, Volume *22*

## Supplemental information

# Efficacious, safe, and stable inhibition of corneal neovascularization by AAV-vectored anti-VEGF therapeutics

**Wenqi Su, Shuo Sun, Bo Tian, Phillip W.L. Tai, Yongwen Luo, Jihye Ko, Wei Zhan, Xiao Ke, Qiang Zheng, Xiaorong Li, Hua Yan, Guangping Gao, and Haijiang Lin**

## Supplemental data

## Figure S1

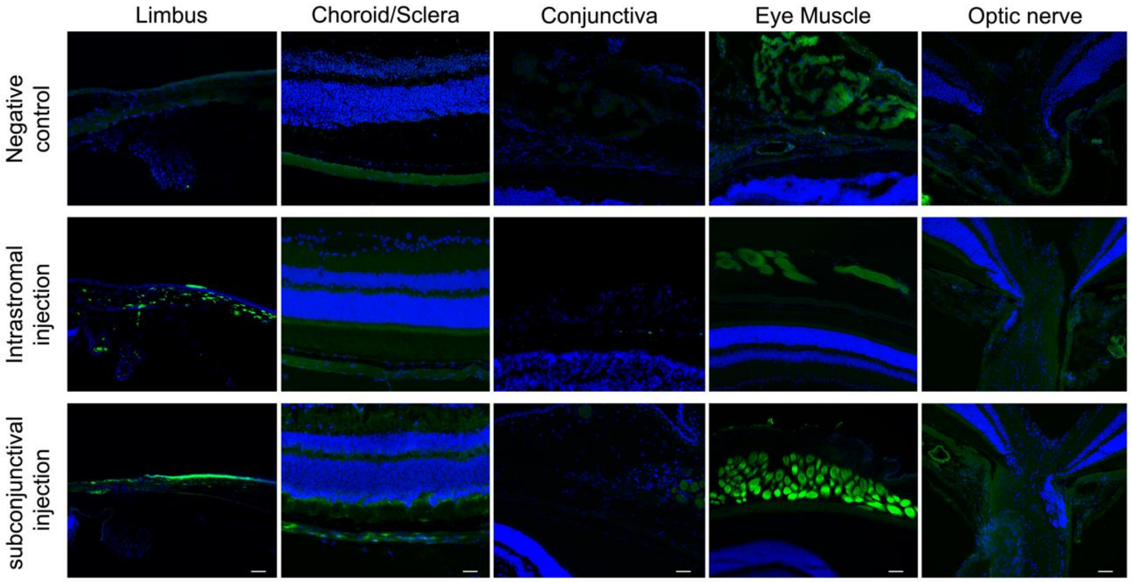


**Figure S1. Representative histologic images of AAV8-mediated eGFP expression and biodistribution in different ocular tissues with intrastromal or subconjunctival injection.** Scale bar, 50µm, 25µm, 50µm, 50µm, 50µm from left to right.

**Figure S2**

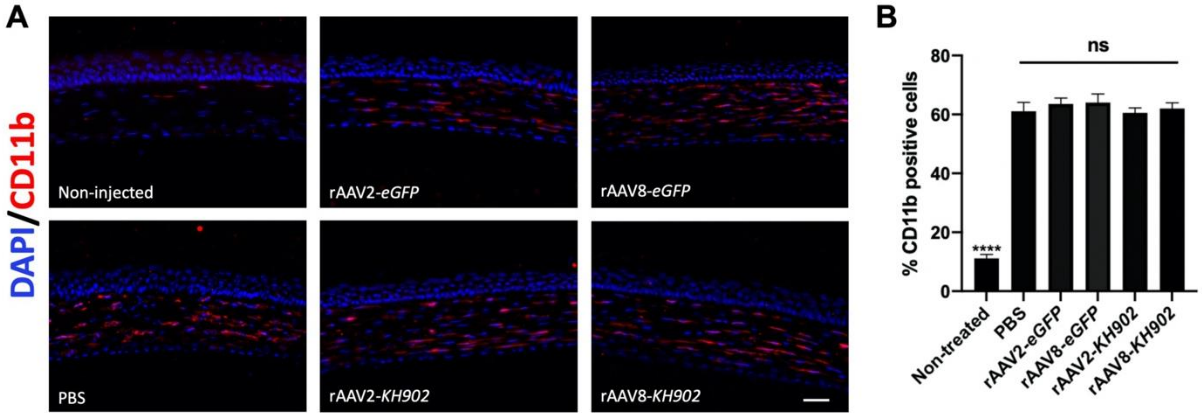


**Figure S2. Analysis of early corneal immune responses following rAAV application. The corneas of mice were harvested at day 4 post injection of PBS or low dose ($8\times10^8$ GCs/cornea) of different AAV vectors individually. (A)** Representative images of immunofluorescent staining for monocytes (CD11b, red) in non-injected, rAAV2-*eGFP*, rAAV8-*eGFP*, rAAV2-*KH902*, rAAV8-*KH902* groups. Scale bar, 50μm. **(B)** The percentages of CD11b+ cells in indicated groups from panel A data. ****, p <0.0001. Data is shown as mean ± SEM, n=5 mice/group.

**Figure S3**

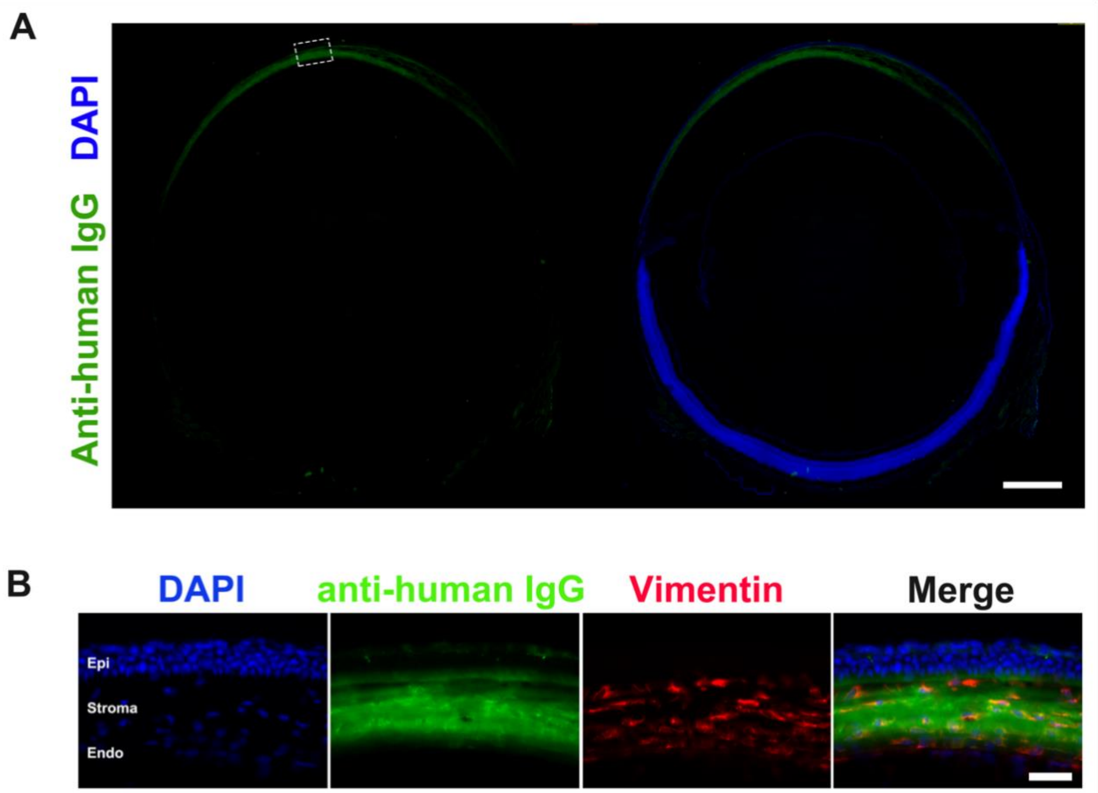


**Figure S3. Histological analysis of KH902 expression mediated by rAAV2 vector in the cornea via intrastromal injection. (A)** Representative eyeball images of KH902 expression marked by anti-human IgG (H+L) antibody (green). Scale bar, 500µm. **(B)** Higher magnification of the boxed regions in panel A with anti-Vimentin co-staining (red), indicating the expression of KH902 was mainly distributed in the corneal stroma layer. Scale bar, 100µm. The dose of rAAV2 vectors was $1.6\times10^{10}$ GCs in 4 µl PBS per cornea.

## Figure S4

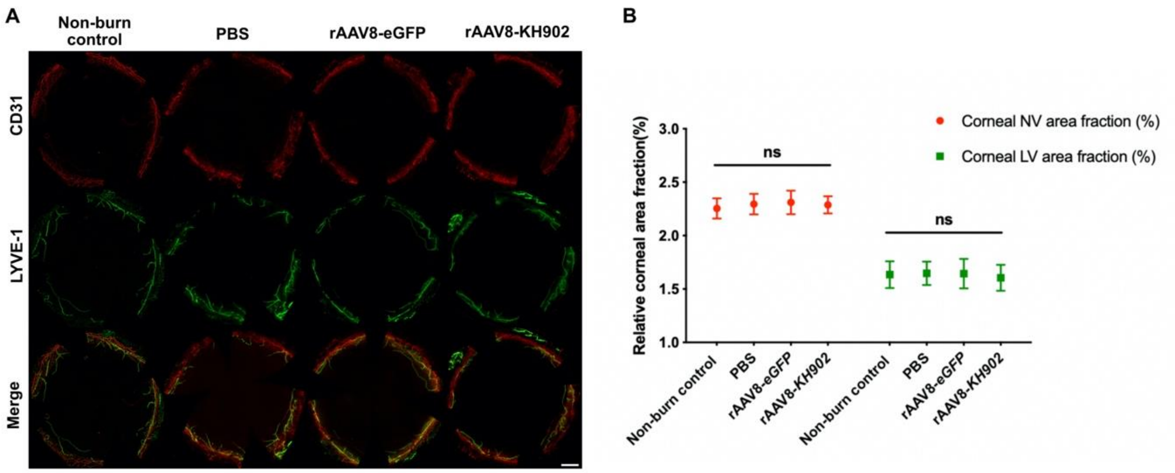


**Figure S4. Immunofluorescence analysis of mouse cornea following intrastromal injection of AAV vectors. (A)** Representative images of corneal flat mount stained with the markers of CD31 and LYVE-1 at 3 months post-intrastromal injection of PBS, rAAV8-*eGFP*, and rAAV8-*KH902*. Scale bar, 500μm. **(B)** Quantative analysis of corneal angiogenesis and lymphangiogenesis by measuring areas covered by CD31 and LYVE-1 staining respectively in each condition of panel A data. Data is shown as mean ± SEM, n=4 mice/group.

**Figure S5**

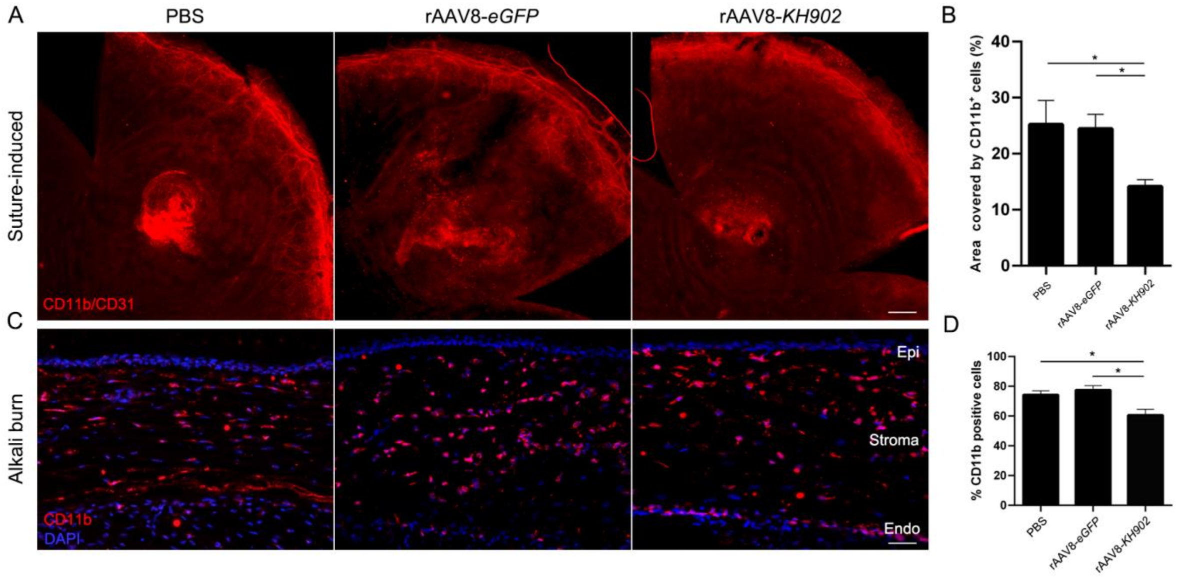


**Figure S5. Inflammatory evaluation in alkali burn and suture-induced models at 2 weeks post treatment**. **(A)** Representative images of PBS, rAAV8-*eGFP* and rAAV-*KH902* groups in suture-induced CoNV model with immunofluorescent staining for monocytes (CD11b) and blood vessel marker (CD31). Scale bar, 250µm. **(B)** Quantification of corneal area covered by CD11b+ cells in indicated groups from panel A data. *, $p < 0.05$. Data is shown as mean ± SEM, n=5 mice/group. **(C)** Representative images of PBS, rAAV8-*eGFP* and rAAV-*KH902* groups in Alkali burn CoNV model with immunofluorescent staining for monocytes (CD11b). Scale bar, 50µm. **(D)** Calculated percentages of CD11b+ cells in indicated groups from panel C data. *, $p < 0.05$. Data is shown as mean ± SEM, n=5 mice/group.

**Table 1.**

**Neutralizing Antibody Titer Following rAAV8 Intrastromal Injection of Burned Cornea**

| **Treatment** | **Mouse#** | **NAb titer** |
|---|---|---|
| PBS | 6266 | No Ab |
| | 6268 | No Ab |
| | 6269 | No Ab |
| | 6370 | No Ab |
| rAAV8-*eGFP* | 6267 | 1/10 |
| | 6374 | 1/20 |
| | 6375 | 1/20 |
| rAAV8-*KH902* | 6265 | 1/20 |
| | 6270 | 1/10 |
| | 6372 | 1/20 |
| | 6373 | 1/5 |
| | 6376 | 1/10 |
